# The Hospital Nursing Supplement

**Published:** 1894-07-07

**Authors:** 


### "The Hospital" Nursing Mirror.

Being the Extra Nursing Supplement of "The Hospital" Newspaper.

[Contributions for this Supplement should be addressed to the Editor, The Hospital, 428, Strand, London, W.C., and should have the word "Nursing" plainly written in left-hand top corner of the envelope.]

### News from the Nursing World.

### LIVING PICTURES.

Many inquiries have been received concerning the possible reproduction of the beautiful photographs which constituted the "living pictures" at the unique limelight exhibition held at the Mansion House on the recent occasion of the Hospital Sunday Fund Conversazione. Some applications for copies have already been received. All who wish for a complete set of these realistic pictures, or for any special copies, should communicate as soon as possible with the Editor, 428, Strand.

### AN OUTSIDE EXAMINER.

The annual examinations of the probationers at the London Hospital have been this year conducted by Dr. Hayward of Haydock, Medical Officer of Health. He expressed himself as thoroughly satisfied with the results of the course of training received by the probationers, forty-three of whom have acquitted themselves successfully. The first prize was gained by Probationer Butteel; Probationers Barnby and Hutchinson taking second prizes, whilst honorary certificates were awarded to Probationers Bickers, Wamsley, Carter, and Beck. The advantages are so obvious of examinations being conducted by doctors other than the lecturers in the training school that it is strange that the plan now adopted at the largest of our hospitals has not long ago been taken up by all institutions.

### INVALID CHILDREN.

A meeting on behalf of the Invalid Children's Aid Association was held on June 29th at the Right Hon. Lord Brassey's beautiful house in Park Lane. The picturesque "India Chamber" was quite crowded before three o'clock, the hour fixed for the proceedings to commence, and late-comers had to content themselves with standing in the gallery or listening from the stairs to the interesting speeches of Sir Charles Freemantle, Dr. Rodgers, Mr. Howard Marsh, and Mr. Timothy Holmes, the Chairman of the Executive Committee, who is also acting temporarily as Hon. Secretary. The illness and absence of Mr. Allen Graham, the energetic founder of the Association, were deeply deplored. Miss Hughes, Superintendent of the Metropolitan and National Nursing Association, gave a charming little address, graphically describing that part of the work of the Association which comes under her own observation in her own district work amongst the London poor. She dwelt especially on the value of visits regularly paid to the delicate or crippled children in their own homes, and spoke of the unconscious influence for good thus exercised over a whole family. Miss Hughes suggested that the time when "everybody" went out of town was a terrible blank for the little child accustomed to "the lady's" visits, and she made an excellent proposal that where any visitor was unfortunately unable to provide a substitute during her absence she should, at least, send an occasional letter or present of fruit or flowers to the little patient as a sign that he was not forgotten.

### THE REGISTERED NURSES' SOCIETY.

The attention of nurses is called to the fact that all nurses who now make application to join this society will be required to pay one guinea as entrance fee. It is greatly to the credit of the Nurses' Co-operation that all such vexatious charges should have been avoided. This latter fact is no doubt due to the circumstance that the Nurses' Co-operation was founded entirely by nurses for nurses, and that those devoted women who originated the movement provided the necessary funds to secure its financial position at the outset.

### OUR NEEDLEWORK COMPETITION.

Most of our readers are contemplating the near prospect of holidays, and therefore the present seems a good time to remind everyone of the work which may well be begun in the comparative leisure of summer days spent in the fair country or by the restless sea. We believe that our constant readers and kindly subscribers might also be the means of spreading knowledge of our Christmas competitions amongst those who have more leisure than themselves. Many persons would surely be found willing to contribute one garment for the use of a sick man or woman spending next Christmas Day within a hospital ward. If every person who reads The Hospital would supply one such useful garment what a glorious collection there would be for distribution. We do not venture to suggest that our male readers should sew, although doubtless some of them are quite capable even of this, but we think if each one supplied enough materials for even one shirt or bed jacket or vest, or gave wool for a pair of socks, a sister or friend would generally be found able and willing to make the desired article. We therefore beg our nurse friends who are so generous and industrious themselves to make our competitions so widely known that the supply of useful gifts sent in at Christmas may go a long way towards satisfying the enormous demand which arises every winter for warm clothes for the sick and poor.

### PRIZE GIVING TO THE WOMEN STUDENTS.

The pleasant garden, as well as the Lecture Hall, at the School of Medicine for Women, was well filled with guests on June 26th, when Lady Lansdowne presented the prizes to the successful students. Speeches were made by Mrs. Garrett Anderson, the Dean, and by Mr. Sheppard and the Rev. S. Paget, the latter, a son of Sir James, giving eloquent testimony to the success which has attended women who, in the face of much opposition, have fought their way into the medical profession, where they have done admirable work. Students, their relations, and many women doctors were present, as well as other ladies and gentlemen interested in this prosperous but unpretentious little medical school. After the meeting had closed with a vote of thanks to Lady Lansdowne for presiding, she was entertained at tea in the garden by the students. The school was inspected by a large number of visitors, and the medical gentlemen especially, cordially commended the admirable condition as well as the arrangements of the different departments. Certainly great credit is due to those who have ingeniously utilised the dwelling-houses at present the premises of the School of Medicine for Women. It is not difficult to foresee that those ladies by whose energy the erection of the beautiful little hospital for women in Euston Road was accomplished will some day have equally appropriate headquarters for their medical school.

### OLD, HELPLESS, AND POOR.

Without any attempt at sensationalism the Commissioner of the *British Medical Journal* has given a report of the workhouse at Haverfordwest, of which the pathos appeals irresistibly to the hearts and consciences of all who read it. Can anyone wonder that the provincial poor-house is dreaded and avoided, whilst the sick and infirm prefer semi-starvation at home to a sufficient diet within those unlovely walls. The Commissioner speaks of plank beds and thin chaff bedding as the existing accommodation for bed-ridden and helpless patients. The attendant, an untrained woman, also looks after the midwifery cases. The sick have no attention and no lights provided during the weary hours of darkness; and the children are kept in a dreary, stone-floored room, of which "nursery" is the most inappropriate title. It sounds like a parody in such a connection!

### NOTTINGHAM.

The annual report of the Nottinghamshire Nursing Association is a very satisfactory one. There are now thirty-six private, nine district nurses, and ten probationers on the staff, and good accounts of their work are received at the institution. The private nursing branch is self-supporting, and £150 1s. was divided amongst the nurses in December, in addition to the fixed salaries received by them. The district nursing is entirely distinct from the private, and is a charity for the free supply of trained nurses to the "sick poor of all denominations, without other qualifications than sickness and necessity, on application being made to the lady superintendent." Many useful gifts have been received by the association during the year for the use of the patients. We are glad to see that all the nurses employed by the Nottingham and Nottinghamshire Nursing Association have had, at least, two years' general training.

### NURSES AT BRADFORD INFIRMARY.

Medals were presented at Bradford Infirmary on the 20th June to those nurses who had passed the examination best, and who were also the most proficient in their practical work. The examiners spoke highly of the standard of knowledge attained to by all the candidates, which had made it a matter of some difficulty to select the prize-winners. The occasion was pleasantly emphasized by the presence of the visiting and resident staff, and also by the matrons of other Bradford hospitals and nursing homes. Dr. Greyder, in congratulating the prize-winners, consoled their less lucky companions by saying that although they had not reached the highest point "they would still make good nurses."

### A GOOD NURSE APPRECIATED.

"He was quite sure that but for her the mortality, instead of 2 per cent. would have been 6, 7, or 8 per cent., for in fever, nursing often turns the scale from death to life," is Dr. Banks' testimony to the work of Miss Robertson, the trained district nurse of the Wick and Pulteney Town Association. There have been 200 cases of typhoid fever since last August, and the low death-rate is noteworthy. The local Press also quotes the late Dr. Alexander's remark, that "he had no hesitation in saying this low rate of mortality was largely due to the skill and care of Miss Robertson." There is little likelihood that the residents in the district where the nurse's services have proved so valuable will fail liberally to respond to the appeal shortly to be made on behalf of the Wick and Pulteney Town District Nursing Association.

### GOOD WORKERS AT BURTON.

The district work of the Burton-on-Trent Nursing Institution has a special point of interest about it, for three night nurses are employed during the greater part of the year, as well as four day nurses, and their services are highly appreciated by the patients. Only efficient trained nurses are engaged, and all their work —which lies among the sick poor—is supervised and each case is visited by the superintendent, Miss Carson. The association possesses a good stock of medical and sick-room appliances, which are most valuable aids in the nursing of many serious cases of illness. Amongst these are enumerated diseases of the heart, brain, kidneys, and spine, nineteen cases of typhoid, the same number of peritonitis, fifty surgical, fifteen paralysis, and many others, all of which have demanded thoroughly competent and skilled nurses, capable not only of attending to the sick, but also of teaching the relations of the patient how to make their dear ones comfortable. The Home for the nurses, which was opened in March, adds greatly to the comfort of the staff. The garden was laid out entirely at the expense of Mr. Salt, the chairman, and the Home, including furnishing, has been completed and left entirely free from debt, thanks to liberal donations.

### THOROUGH.

Our contemporary, *The Trained Nurse*, has much interesting news this month regarding the work of the American Nurse Training Schools. It reports the reception at the Tracy Home, Des Moines, Iowa, of a photograph of Miss Florence Nightingale endorsed with her signature and the following lines, "I can say in every way adieu. Thorough, thorough, thorough! God speed everyone who is thorough."

### SHORT ITEMS.

Five thousand pounds is needed to put the Seaford Convalescent Hospital in a satisfactory financial position: 1,058 patients were received at the hospital during last year, with the result that the income did not cover the expenditure by £650.—Eight male nurses have died of typhus at the Mustapha Hospital, Algiers.—*Nursing Notes* for July contains a useful paper on "The Nursing of Epileptic Cases"; and in the Report of the District Nursing at the Central Home is given an excellent idea of the important and serious cases which come within the experience of the trained district nurse. The Marchioness of Bute is the Hon. President of the recently established Rothesay District Nursing Association. — A garden party was recently given to the nursing staff of the West London Hospital by Mr. William Bird, J.P., D.L., chairman of the committee. The weather was lovely, and the afternoon was a most enjoyable one to all present.

### On General Nursing.

By Rowland Humphreys, M.R.C.S., L.R.C.P.Lond.

XIX.—PERITONITIS—(*continued*).

Inflammation in the right iliac region is another common cause of peritonitis. The inflammation in this part of the abdomen is said to be due, in all cases, to injury to the vermiform appendix. This little tube is closed at one end, at the other it opens into the intestine at the cæcum. It is a "survival," and does not appear to be of any use in the human body. It sometimes becomes plugged with a hard little mass of fæces, or with mucus, and, ulceration taking place, the ulcer may, and often does, set up the formation of an abscess outside the intestine, perhaps without any direct opening into it from the intestinal canal. This abscess tends to rupture into the intestine, often finding its way down into the pelvis and opening into vagina or rectum; or it may open into any other part of the intestine, or externally, or into the peritoneal cavity. In the last case, if the operation can be done early enough, the abdomen has to be opened, washed out, and drained. These cases of appendicitis are extremely dangerous to the patient, and are very anxious to all concerned. In perityphlitis, as a rule, the peritonitis is at first localised, confined to the neighbourhood of the inflamed part, but gradually extends, a hard, well-defined tumour, due to the pent-up contents of the abscess sac, making itself perceptible to touch and percussion. As soon as the abscess bursts the patient feels better, provided it has not ruptured into the peritoneal cavity. In this case severe pain and shock is experienced, and if unrelieved, the patient rapidly dies.

The treatment of such cases resolves itself into relieving the pain, and with precautions causing the bowels to act. The action of the bowels relieves the patient greatly and makes him more manageable, but remembering that the safety of the patient is alone assured by the formation of adhesions between the inflamed and the surrounding organs, it will be understood that any violent movement of the bowels is to be carefully guarded against. On the other hand the administration of opium is, except as morphia subcutaneously in sufficient doses to relieve pain, to be equally strongly deprecated. It appears that eighty years ago a well-known medical man advised, in cases of peritonitis, that the patient should be kept from movement and from food, and that he should be given purgatives, have cold applied to the abdomen, venese action to be sparingly used, and opium very rarely. The practice of administering was, till quite recently, the one treatment for peritonitis, but Mr. Lawson Tait revived the older methods just mentioned, and we now recognise that many cases are very satisfactorily treated by the administration of a purge at the commencement, and sometimes at a later stage of the complaint. In perityphlitis the writer has for some years made use of purgatives, in the form of castor oil and opium, as many of the cases appeared to be suffering from the effects of absorption from the intestine, and the cases all did most satisfactorily, yet there is undoubtedly a danger in the treatment.

Perityphlitis is recognised by the onset of severe pain in that part of the abdomen where the cæcum lies, by the skin becoming very tender there, with the pain often shooting down the leg, and not uncommonly with some symptoms of bladder irritation from the extension of the disease to it. The pain increases, and with the onset of peritonitis it becomes very severe, radiating towards the umbilicus, and is accompanied by vomiting, &c. The formation of an abscess is known by the usual signs, the temperature rising rapidly, and varying a degree or two only night and morning, being preceded by a rigor, and accompanied by signs of the presence of matter at the seat of the tenderness.

Idiopatic or simple peritonitis, that is, peritonitis caused by something which we cannot exactly define, is especially likely to attack persons suffering from heart or kidney disease. It also occurs in people who have had a previous attack. In these cases an operation can only rarely be advisable. In cases having an attack for the second or third time the adhesions are usually so considerable that nothing can be done when the abdomen has been opened. The object of opening the abdomen in such a case would either be to discover the cause of the disease, or to empty the products of suppuration. In the first case an operation would probably be very difficult, and the numerous adhesions would render the operation so prolonged, and the danger of bleeding from the vessels which would have to be divided in the process so considerable, that it would most likely have to be abandoned, whilst the pus, &c., could not be properly cleared out for the same reasons. Operative procedures in chronic cases are therefore rarely done, and still more rarely successful. Not long ago the author had a case which came into the chronic category. The patient, an elderly woman, had had attacks of peritonitis on and off for some twenty-five years. All parts of the intestines were matted together and thickened, and the end came when, from the contraction of these thickened membranes, a part of the bowel was compressed, death ensuing partly from obstruction, partly from peritonitis.

Women frequently suffer from peritonitis of the pelvis proceeding from some inflammatory condition of the ovaries or, more frequently, of the fallopian tubes, the little ducts which convey the ovum or egg from the ovary to the uterus or womb. Unfortunately these tubes act as paths along which the disease may spread from the uterus and vagina upwards to the peritoneum, into whose cavity they open. This is a common cause of the pelvic troubles to which women are liable. The peritonitis is usually confined to the pelvis, and only rarely spreads higher. This last happens, however, in all very virulent conditions, as in that form of septic trouble which is known as puerperal peritonitis immediately following the birth of a child. This form, in common with most other forms of the complaint, is very infectious and very fatal. When the suppuration is localised, and does not of itself open into rectum or vagina, as is usually the case, an operation may be performed for its relief. Sometimes it bursts into the peritoneal cavity, causing rapid death.

### Minor Appointments.

Royal Infirmary, Hull.—Miss Sophia Coulthurst, who was trained at the Liverpool Royal Infirmary, has been appointed Night Superintendent at Hull. Miss Coulthurst acted as Night Superintendent and Charge Nurse at the Liverpool Royal Infirmary, and her record in the register of the school is very good. She was afterwards Parish Nurse at Bebington, Birkenhead, for three years, and all her testimonials prove most satisfactorily that Miss Coulthurst takes many kind wishes with her to her new post.

Northern Hospital, Winchmore Hill, N.—Miss L. A. Morgan, who was trained at the Royal Infirmary, Edinburgh, has been appointed Matron of this hospital. She was Sister for some time at St. Marylebone Infirmary, and has for the last two and a half years held the post of Night Superintendent at the London Fever Hospital. Miss Morgan has excellent testimonials, and the experience gained in doing temporary Matron's duties, which she has accomplished most satisfactorily, have shown her to be an excellent manager. We wish her equal success in her new post.

### The Mechanism of Movement.

VII.

Why do joints work so easily and never give us any pain? In a fresh joint its appearance in life can be readily studied. In the ball and socket joint the round end of the bone, as well as the cup, are covered or lined with a smooth substance called "cartilage," or "gristle" kept moist and smooth with synovia. Cartilage contains no nerves, and has no feeling; if it had, we should have pain when we moved. The bones are kept in place at the joints by very strong bands or ligaments; in hinge joints a number of these bands are fastened above and below, but in ball and socket joints they also surround the joint, forming a cap, in which the joint moves freely. In disease this smooth cartilage gets worn away, and the ends of bone rub together like those of a skeleton; the pain is great, because the bones have nerves, though the cartilage has none. A bone without cartilage is like a decayed tooth with an exposed nerve. In a healthy tooth the nerve is well covered, and gives no pain; and in a healthy bone the nerves are there, but they are only felt when the cartilage is worn away. This is why hip disease is so painful. Either the cartilage is worn away or the synovia used up, and the joint moves with difficulty, like a rusty hinge, with great stiffness and pain, or sometimes stiffness alone without pain. Hip disease occurs chiefly amongst children. They are restless, never still for long together, and more likely than adults to get some blow or injury, which is forgotten at the time, but may have serious consequences. The hip-joint, because capable of so much movement, does most work, is liable to most injury, and is most often the seat of disease. The hip-joint is well surrounded by thick coverings of fat, sinew, flesh, and skin; but although out of sight, when disease gets hold of it, it gives decided warnings of its existence, and a watchful person easily recognises the early signs of disease. When severe pain occurs at a very early stage which cannot be overlooked, proper medical advice is obtained.

We must now speak of the muscles. What we call "meat" is synonymous with certain muscles of the cow or sheep.

Some doctors say much can be learnt of a person's health by feeling the arm and noting whether the muscle feels soft and flabby, or firm and solid. Also, walking behind people in the street one doctor says he has noted whether people's cheeks shook as they walked, and he always found, in his experience, that flabby muscles meant indifferent health.

Muscles are for the most part spindle-shaped, that is, narrower at either end and thicker in the middle; and when they contract to bend a bone, they become shorter and thicker. It must be remembered that muscle is governed by the will through the nerves. Where the muscles pass over joints they become harder, flatter, and broader, and are then called "tendons," or "leaders," and the reason for this is that the muscle may have a firmer hold, and not be torn off in violent exertion. The tendons are very strong, their function being to join the bones and the muscles, and they are probably the strongest tissue in the body.

It is very rare indeed to find tendons actually torn, though they can be strained, and very painful they are then, far worse than a broken bone.

In every muscle one end is more or less fixed, called the "origin," and the other end is joined on to a bone that moves, and is called the "insertion." As the muscles are intended for motion, if they are not exercised they become weak, soft, and actually smaller, as is seen in a bed-ridden person. There are about 400 muscles in the human body, and these form the "flesh" or "lean" of the body. If we look at a piece of meat cut across the middle, we see that the red flesh does not form a uniform mass, but is divided up into unequal and variously shaped areas by white lines, forming partitions. Each of these areas is a separate muscle, and if followed upwards and downwards will be found to end at either end in a sinew or tendon, which attaches it to the bone. To enable the muscles to do their duty we must have some governing centre. We contract our muscles without effort and without exercising at each movement a distinct mental wish. We should find it very embarrassing had we, when walking, to make a distinct mental effort to place one leg alternately before the other. This central governing power is the brain, from which innumerable nerves proceed to each part of the body, and branches enter each muscle bringing it under control. The act of walking brings almost every muscle into play, either directly for progression, or indirectly for balancing the head and trunk. The muscles of the arms are the least concerned, but even these come instinctively into action to some extent. The chief muscles concerned in walking are those in the calf and back of leg, which by pulling up the heel, also pull up the bones of the foot connected with it, and then the whole body, the weight of which is passed on through the bones of the leg. When walking the trunk is thrown forwards so that it would fall down prostrate were not the right foot planted in time to support it. The calf muscles are helped in this action by those on the front of the trunk and legs, which contract and pull the body forwards, and the trunk slanting forwards when the heel is raised by the calf muscles, the whole body will be raised and pushed forwards and upwards. This advancement of each leg is effected partly by muscular action, the muscles used being (1) those on the front of the thigh, bending it forward on the pelvis; (2) the hamstring muscles, which slightly bend the leg on the thigh; (3) the muscles on the front of the leg, which raise the front of the foot and toes, preventing the latter, in swinging forwards, from hitching in the ground. When one foot has reached the ground the action of the other has not ceased.

There is another point in walking. The body is constantly supported and balanced on each leg alternately, and, therefore, on only one at once. Hence there must be some means for throwing the centre of gravity over the line of support formed by the bones of each leg as it supports the weight of the body. This is done in various ways, and hence the differences in the walk of different people. There may be slight rotation at the hip joint, bringing the centre of gravity of the body over the foot of this side. This "rocking" motion of the trunk and thigh is accompanied by a movement of the whole trunk and leg over the foot planted on the ground; and is accompanied by a compensating outward movement at the hip. The body rises and swings alternately from one side to the other as its centre of gravity comes alternately over one or the other leg, and the curvature of the spinal bones is altered with the varying position of the weight.

### A Case for Assistance.

Miss Bell, of the Liverpool Homes for Aged Marines, writes to say that in response to the appeal made in The Hospital of June 30th on behalf of a nurse, she has received the following sums:—10s. from Miss F. C.; Dr. J. B., 10s., and Miss Y., 5s.

### Presentation.

On leaving the National Sanatorium, Bournemouth, Miss Gertrude Ridgwell was presented with valuable presents from the Committee, Chaplain, Lady Superintendent, and patients. Miss Ridgwell, who leaves to be married, takes with her the respect and good wishes of all who have come in contact with her during the four year she has been nurse to the female patients at this institution

### The Royal Visit to the British Home for Incurables.

Streatham was gaily and prettily decorated on Tuesday and early in the day crowds of spectators began to traverse the route which was taken by the Royal party, who arrived at the new Home before the clocks had finished striking four. The sight of the first carriage with its four horses and outriders in scarlet was the signal for prolonged cheering without and within the gates.

The Princess of Wales, "Our Princess," looked fairer and younger than ever in her graceful black dress, relieved with white vandyke lace collar and a dainty bonnet, forming a restful and gracious picture amongst the many gorgeous colours affected in the fashions of the day.

However appropriate the youth of Cambridge may have found the title of grandfather in the hearty congratulatory greeting they gave to the kindly Prince the other day, the corresponding word grandmother seems the last one likely to enter into the mind of anyone gazing with loving respect on the Princess. On the arrival of the Royal party on the terrace the Archbishop of Canterbury offered up special prayer, after which a number of ladies and children presented purses to the Princess of Wales. The obvious difficulty of retreating backwards down several stone steps caused considerable amusement.

On the whole, the feat seemed to be accomplished more easily by the children than by their elders, who in many cases turned their backs on the Royal party whilst looking for a safe foothold, and those who had long dresses anxiously glanced round and arranged them preparatory to retiring in the fashion demanded by Court etiquette.

In the absence of Mr. Beavan, Mr. Hale, the treasurer, read the address :—

May it please your Royal Highness,—The board of management of the British Home for Incurables desire to express their sincere gratification that on this auspicious occasion of the opening of their new Home they are honoured by the presence of your Royal Highness and of his Royal Highness the Prince of Wales. The institution was founded in the year 1861, and commenced operations with a collected sum of £3,743. In the year 1863 your Royal Highness became patroness of the institution, the first charity in the kingdom on which your Royal Highness was pleased to bestow this mark of special favour, and since that time it has constantly been the recipient of your Royal Highness's thoughtful sympathy and assistance. The institution has now an income of £19,705; the number of pensioners, each receiving £20 per annum, is 288, and there are at present 44 inmates of the Home. Being compelled to vacate the premises in Clapham Road, owing to the expiry of the lease, the board determined to build a home containing every modern convenience for the comfort of the inmates, and after long deliberation selected the site on which this house, about to be opened by your Royal Highness, has been erected. The total cost, exclusive of the chapel, which we owe to the liberality of Miss Leicester, has been £27,500. Towards this amount £22,200 has been contributed, and it is confidently expected that the friends of the institution and the public will so appreciate your Royal Highness's kindness in consenting to inaugurate the new Home, that the balance remaining will be at once cleared off, so as to leave the existing funds available for the maintenance of the institution. The board, on behalf of the subscribers and those present, take this opportunity of offering their heartiest congratulations to your Royal Highness and his Royal Highness the Prince of Wales on the birth of a grandson, and trust that your Royal Highnesses may enjoy many years of health and happiness, and continue to assist with your sympathy this and the many other charitable institutions of which your Royal Highness is the patroness.

To this the Prince of Wales made the following reply :—

I am asked by the Princess to return her warmest thanks for the address we have just heard, and for the welcome given her to-day. I am also to tell you the great pleasure it has given her to take part in this day's proceedings and in the opening of this new building for so admirable an institution as the British Home for Incurables, of which she has now been patroness for thirty-one years. It is impossible to conceive a more charitable or a more excellent object than to help our unfortunate fellow creatures in their great suffering, and although they may know that they cannot ultimately recover, they have at least the advantage of competent nursing and fresh air, and of being well cared for to the end of their lives. It must be one of the best philanthropic objects in which anyone can possibly engage. I must also add a word on my own part. I think I may safely congratulate you on the fact that, with so heavy an expenditure, through the great liberality of so many, you are now free from debt. I only hope you may continue so, and that you may long continue to flourish.

After the unveiling of the Princess's Coat of Arms, which was carved on the stone of the building, the formal proceedings ended, and the Royal party payed a prolonged visit to the Fair in the rear of the building, entering each of the shows and staying for a short entertainment in each. Their Royal Highnesses the Princesses Victoria and Maud accompanied their parents, attended by Lady Emily Kingscote, Lord Colville of Kinross, and Colonel Clarke.

The British Hospital for Incurables has been a familiar landmark in the Clapham Road for so many years that it seems strange to think of it in any other locality.

The site of the present building is certainly a happily-chosen spot, for it stands on high ground in the midst of very pleasant surroundings. The sitting-rooms have large windows, which give the patients an excellent view from every portion of their airy apartments. The wards all contain five beds, and a composite piece of furniture standing beside each, provides a fixed looking-glass, with washstand and chest of drawers. The light bamboo screens have washing covers, and the rooms are all warmed with hot air, but have fire places also. The lavatories and bath-rooms are all built on the turret plan, and are complete on every floor. There is a convenient lift roomy enough to convey an invalid couch when it is necessary or desirable to convey a patient from one floor to another.

The nurses' bed-rooms and sitting-room are at the top of the house, and the latter is sufficiently large to make the fact of its position just under the roof seem a secondary matter. It possesses a charming view of fields and hills, and is simply and substantially furnished. We were told that each nurse has a separate bed-room, but there did not appear to be one open for inspection on Tuesday, which was a disappointment to those interested in the housing of nurses. The basement, in which are situated the kitchens, store houses, &c., is very spacious and light and airy withal. The bath-chairs for the patients' use in the grounds have a conspicuous place, conveniently near to one of the exits.

The "fair" was held in a field at the back of the Home, a lot of "market booths" being presided over by a distinguished company of ladies. The shows offered considerable attractions, and included a theatre, Richardson's Show, variety entertainments, tableaux vivants, and a concert tent. The Executive Committee for Theatrical Entertainment for the Endowment of an Actor's Bed, with Sir Augustus Harris for chairman, was composed of many well-known actors and actresses, but their chances of securing a substantial endowment must have been considerably curtailed by the fact that the majority of visitors present had tickets which entitled them to free admittance, so that the performers who had so liberally given their services were here as elsewhere victimised by influences of "paper."

Mr. Gofton Salmon, the energetic secretary, is certainly to be congratulated on the successful arrangements made for this large and brilliant gathering.

### Notes from Victoria.

(Communicated).

### A CHEERING OUTLOOK.

Notes sent from time to time about Melbourne and Australia generally must have given The Hospital readers an impression that we have enormous areas of land available for stock or cultivation, that our gold and silver mines are wonderfully productive, that in fact we have untold riches. Riches have been gathered in fabulous quantities years ago, and some have been scattered and wasted most wantonly. We have not yet learned the art of self-government or of wise expenditure. But we are learning, and the wealth of the country is still great and waiting development.

We shall be sorry indeed if people in England are turned aside from their belief in this by our recent unparalleled "depression," or by the ill-judged appeal for money for our Melbourne poor made in the *Times* by Professor Morris. Our poor are not in such straits as he supposes. The Rev. H. Tucker (the author of the New Arcadia), Dr. Strong, and others are making noble efforts to settle men on the land in small holdings of ten or twenty acres. Obstacles have to be overcome in order to make this a success, the worst one being the complete idleness of some of the men—when they find that only industry will bear good fruit they are discontented and fault-finding.

Too much indiscriminate giving of charity in Melbourne for years past has attracted men out of work from all the colonies, and this aggravates our situation; but as the Premier very truly said when he sent the cable to our Agent-General: "It is certainly not the case that the resources of the colony are exhausted, or that the springs of benevolence have run dry. On the contrary, things are steadily improving, and there is every prospect of complete recovery from recent temporary troubles. Perfectly able to deal locally with wants of distressed."

Bullarto, of typhoid fame, is not yet clear of the epidemic, or Tylden of diphtheria. Bullarto stands 2,452 feet above the sea; Tylden 1,884 feet. There is no excuse for diseases of this character. The two places are on the line of railway between Woodend (a town strip on the side of Mount Macedon) and Daylesford. The journey is through very lovely scenery; no tourist will have seen the healthiest and most beautiful part of Victoria if he does not visit Daylesford. The trade of wood-cutting has been going on for many years all along this line, and may go on for many years more without any apparent diminution of trees. Potato growing is extensively carried on where the ground is cleared, especially at Tylden, Trentham, and Lyonville. Daylesford town is on the crown of a hill; it is most picturesque, has its town hall, convent, Roman Catholic church, English, Presbyterian, and other churches, and a substantial hospital with a separate fever ward in its grounds. An old resident has just left £1,000 to be used for the building of a women's ward. The views from every part of the town are extensive and magnificent. At sunset the ranges of the hills in the distance with the beautiful purple hue; further off, hills of a blue hue, seeming to touch a golden sky, are lovely beyond expression. On leaving the town on the Castlemaine Road the nearer wooded hills have the Australian characteristic of silence, which in the woods is awe-inspiring, and must be felt to be understood.

There are some pyrites works in this vicinity, said to be the "finest in the colony," and beyond stands the Italian house of one Holleri—years spent in this country still leave him an Italian, but his children are quite Australian. In a ravine in front of the house are the famous Hepburn Springs. The water is delicious and very beneficial, but is not nearly sufficiently appreciated. In the winding of the "creeks" men are found "fossicking" and "prospecting" for gold. Much has been got, and is still to be got. Not long ago two men, a few miles further from Holleri's, got gold to the value of £800, working in their claim from Wednesday to Friday in one week. They and others have found more since. No, we are not a *poor* colony yet; but we are not wise when we ask for money from our mother, England, where working men and women have not half the chances we have for making it.

### Nursing in Germany.

A military surgeon, Dr. Keyse, of Berlin, is strongly urging the construction of a greater number of portable hospitals for use, not only during epidemics, but also in time of war. He mentions the lamentable want of hospital accommodation in Hamburg during the cholera epidemic to show how necessary and important it is to have a sufficient number of such hospitals in readiness in case of need. During the war between France and Germany in 1870 they were found invaluable, and everything medical and surgical is now usually constructed with especial provision for the exigencies of the battlefield. At the Hygienic Exhibition at the International Medical Congress recently held at Rome the portable hospitals of the Prussians received the highest commendation from doctors, those in general use providing accommodation for twenty patients, and containing a dispensary, waiting-room, bathroom, operating-room, and kitchen. They can be put up in a garden, or on any small plot of ground, by quite inexperienced persons, owing to the simplicity of their construction. Both the deceased Empress Augusta and the present Empress have done much to improve the condition of hospitals. The former offered prizes for the best portable hospitals shown at the Antwerp Exhibition in 1885. The present Empress of Germany was instrumental in procuring one erected at Tempelhof, Berlin, in 1891. According to the half-yearly report issued by Dr. Meerger, 273 patients have been treated in this hospital from July 1st to December 15th, 1892. It contains three wards and special accommodation for the physicians and nurses. Two differently constructed hospitals, designed respectively by Doecker and Bernhardt-Grove, are now in use in German garrisons, though Doecker's seems to be the favourite, 71 of that make having already been erected in Prussia.

The German Women's Society for Colonial Nursing has formed new branches in Düsseldorf and Dessau, making in all fifteen branches. Frau v. Scheele has been appointed head of the Zanzibar branch. The society obtained the services of seven sisters from the Clementine Nursing Home in Hanover for special work in East Africa. They rendered excellent service, and now that all danger of war in East Africa has subsided they have returned home. Two Sisters from Mannheim have offered themselves for work abroad, and will probably leave Germany for Togo in the autumn.

Messengers calling up medical men at night need no longer spend anxious moments of suspense at the house door in uncertainty as to the doctor having heard the bell or being at home. Herr Költzow is the inventor of an apparatus to be fixed outside the house, beside the night bell, and connected with the doctor's room. Immediately on hearing the bell the doctor sets the apparatus in motion, and on the top slab of the apparatus, composed of glass and illuminated by means of a small lamp, appear the words "ich komme.—Dr. N."

Complaint is often made at hospitals about the milk not being delivered in a perfectly pure condition. Herr A. Bernstein, of Frankfort, advocates the transport of milk from large dairies, well known to be free from all disease, by rail in a van specially constructed and heated, so that the milk is kept at an even temperature of 70 degrees C. This system of supplying milk slightly increases the expense, but has been successfully carried out in many places during the past two years. Herr Bernstein first stated his ideas on this subject at a lecture delivered in connection with the German Society of Public Health.

### Private Nursing in Connection With a Hospital.

In a previous contribution attention was directed to some of the effects resulting from the connection of private nursing with a hospital, and to the advantages and disadvantages to the parent institution.

The subject should also be considered from two other aspects, one from the point of view of the advantages derived by the nurses themselves from their continued connection with the hospital, the other which deals with the benefits which the public may expect to obtain from the employment of nurses attached to a hospital.

There cannot be a doubt that the personal knowledge of a competent and thoroughly well-trained woman is necessary for the proper selection of trustworthy and capable nurses; and a true estimate of their several values can only be made by a sagacious matron who has had the nurses under her supervision for a considerable period.

No amount of written testimonials can be relied on to give perfectly satisfactory evidence of special qualifications for private nursing in regard to the selection of women as private nurses, and unless the greatest care is observed in sifting and weighing testimonials, paper characters of nurses are not likely to be more valuable than paper characters of servants; and most householders know the danger of referring to servants' registries where servants are recommended upon the strength of written communications.

Because a woman has been under training for a certain period, because she has received a certificate of such training, it by no means follows that she will prove a trustworthy private nurse; and institutions which propose to send out nurses without personal experience of their knowledge and character cannot, in my opinion, compete satisfactorily with those private nursing institutions which are attached to a hospital where the nurses are not sent out as private nurses until they have been for some time under the eyes of the matron; and the hospital will have even greater advantage if the nurses employed on the private staff have been trained at that hospital from the very commencement of their career. Private nursing in many ways is so distinct from hospital nursing that it is evident that special qualifications are necessary for a good private nurse, and I have known many excellent hospital nurses who, from some reason or other (it may be a matter of education, manner, or temper), are not fitted for private nursing. Some women will work extremely well under the direction of a competent sister, and while subject to the discipline of a hospital, are ill-qualified for undertaking a post in which they must depend upon their own resources and act upon their own knowledge and responsibility. Not only is great experience necessary to enable a private nurse to act reasonably upon an emergency, but also great tact is requisite in dealing with questions of every-day occurrence in a complex household.

But also it can hardly be doubted that a private nurse should be selected with a due regard to the special demands of education and intellect of the patient who requires her services.

Anyone who has had personal experience in time of sickness of the habits and customs of women brought up among the lower class and accustomed to deal only with the class of hospital patients, knows very well how often the whole behaviour of the woman jars upon the susceptibilities of a refined and intellectual patient.

The way in which a woman of this class moves about the room, her carelessness in regard to personal niceties, her conversation, and the various little details of nursing in which she gives offence—all these peculiarities are often constantly irritating the patient; and in institutions where the names of the nurses employed are entered on a list, and the order of rotation is strictly observed, it is unlikely that the particular needs of the private patient will be as carefully attended to as they would be in a hospital institution, where a careful selection of the nurse best suited to the case is made by a matron, who has personal knowledge of the several nurses under her charge, and some insight into the class peculiarities of the patient who requires nursing.

It seems evident that the skilled selection of a special nurse for each case, which ought to be made, and can be made by the hospital matron, is much better for the public service than the order of rotation, which must be observed by institutions which have to see that all their nurses are kept in employment on equal terms.

The next point which may be urged in favour of attaching the private nurse to a hospital is a very important one; it is this, that the nurse so attached has always the opportunity in the interval between one case and another of keeping up her knowledge by working in the hospital wards as a reserve nurse or a holiday substitute, and by attending any lectures that may be given by members of the hospital staff.

The nurses themselves are quite alive to the necessity of keeping up their knowledge by such means, and I have had nurses who objected, upon this ground, to be more than a certain time with a chronic case.

The advantages, then, which may be derived from the connection of a private nursing business with a hospital may be set out as follows: The hospital can thus retain a hold on the services of well-trained and trusted women who have been under the supervision of the matron, and they will form a reserve from which any deficiencies in the staff can be made good.

The nurses have the advantages of a home, to which they may be reasonably expected to be attached—a home to which they may have recourse if they are ill or out of work. They will be able to avail themselves of the knowledge to be derived from hospital work and from lectures given by the medical staff. Their employment will depend upon the reputation of the hospital, and the practice of the medical staff, which is being constantly renewed by the succession of the younger men, and which is not likely to diminish.

The public will be supplied with nurses who have been carefully selected from personal knowledge, and who have been under supervision for some time, and they may have confidence not only that the character of the women sent out is good, but that their knowledge is maintained up to date, and that a selection of the most suitable nurse for any case is made by a competent and well-trained matron. R. E. T.

### Notes and Queries.

### Answers.

(94) *Nursing Homes (Nurse B.).*—See *Burdett's Hospital Annual*, published by the Scientific Press, 428, Strand, for addresses required. If you write to these you can ask the superintendents to answer your question as to accredited agents.

(95) *Needlework (Tired Worker).*—Write particulars as to time and place where you want the work done, and address your letter to "Needlework," care of "Nursing," Editor of The Hospital, 428, Strand. Probably a lady who does just what you require will be able to undertake fresh work next week.

(96) *Matron or Caretaker.*—The note signed "Nurse" and accompanied by no other name or address cannot receive attention. If our correspondent will read our rule, she will know this, and if she will give her address she sha l have the stamps contained in her letter returned at once.

(97) *Training (A Youthful Aspirant).*—Get "How to Become a Nurse," by Honnor Morten. Pub. Scientific Press, 428, Strand.

(98) *Private Nurse (Convalescent Letter).*—Let us have the name and address of the poor man, and tell us if he can walk.

### Queries.

(94) *Nursing Homes.*—Will you kindly tell me what nursing homes there are at Cannes and Nice, and also whether they have any agents in London?—*Nurse B.*

(95) *Needlework.*—I shall be most grateful to anyone who can tell me of a lady who would come and do all kinds of mending and renovating at my own house. I have no time to put in the occasional stitches my clothes require, with the result that I am driven to buy new ones oftener than I otherwise should.—*Tired Worker.*

(96) *Matron or Caretaker.*—Information asked.

(97) *Training.*—How and where can I be received for training as a nurse?—*A Youthful Aspirant.*

(98) *Private Nurse.*—Can you help me to get a letter for a convalescent home for a poor man who has had an accident and is not yet able to return to work?—*Convalescent Letter.*

### Two Hours Off Duty.

AROUND GUY'S HOSPITAL.—II.

The curious "token books" of Southwark parish contain the records of all persons dwelling therein over the age of fifteen, with a view to the infliction of fines for non-compliance with the statute requiring the reception of the Sacrament. In 1623, sixteen players are mentioned as having partaken of Communion at St. Saviour's; the church is, indeed, haunted by memories of actors and playwrights; Fletcher, Massinger, and Shakespeare's younger brother Edmund, are buried here.

The tomb of a famous quack who lived in the reign of Charles II. will interest nurses; he sold pills, and his monument states that their merits—

"Will survive his dust, and not expire
Till all things else at the universal fire!"

Mrs. Piozzi dwelt for some time in the Borough during the life of her first husband. She rather complains that she was compelled to live in houses she had not first inspected. "When winter came" (1763), she writes, "I was carried to my town residence, Deadman's Place, Southwark;" to this house Johnson was, of course, a frequent visitor. On May 25th, 1780, he wrote, "Congreve, whom I dispatched to the Borough . . . is one of the best of the little Lives (of the Poets), but, then, I had your conversation!" The doctor wrote the advertisement in which the widow offered Mr. Thrale's brewery for sale, and no better example could be quoted of the Johnsonian style. "We offer not tubs, nor vats, but a potentiality of wealth beyond the dreams of averice." Mrs. Thrale had, however, quitted Southwark l efore this for the more "genteel" region of Grosvenor Square.

Mrs. Piozzi prided herself upon having successfully traced the origin of a curious sign on a Southwark house: "The Old Pick-my-Toe" had reference to a Roman slave taking a thorn from his foot.

In one of Johnson's letters to this lady he gives an account of the Lord George Gordon Riots, when the Marshalsea Prison in Southwark was broken open, and the prisoners were released.

We all remember the immortal "Boots" of the White Hart Inn in Southwark. Unfortunately, the building was pulled down in 1889, but Sam Weller's inn was the successor to an older one, wherein Jack Cade held his Court during the short supremacy of the rebels.

When Southwark Street was altered, distinct evidence was found that "Lake Dwellers," inhabiting houses built upon piles, once lived in this neighbourhood. Remains of Roman villas have been discovered at various times, and amongst these, no doubt, the Saxons formed their village, which William the Conqueror thought it prudent to destroy.

Little Dorrit's home in Southwark was called the Marshalsea because "pertaining to the Marshals of England." Littleton, the famous lawyer, was judge of the Marshalsea Court during the reign of Henry VI. A prison under the jurisdiction of the Marshals existed here in the reign of. Edward III., and the Kentish rebels destroyed it in 1381. The Marshalsea of Dickens stood in the High Street Bonner, Bishop of London, died in the prison during Elizabeth's reign. He was buried secretly in the churchyard of St. George's, Southwark.

It is interesting to note that the first English Bible "was imprinted in Southwark for James Mylson" in 1536.

The list of dwellers "in the Liberty of the Clink," or within that prison itself, includes the names of Alleyn, the actor-founder of Dulwich College; Philip Henslow, who was a stage manager and "master of the bears" in the reigns of Elizabeth and James I., and Haughton, the dramatist.

The minutes of Privy Council meetings in the reign of Mary were frequently dated from the Bishop of Winchester's house within the Clink Liberty.

About the middle of Queen Anne's reign the High Church party began once more to assert the old doctrine of passive obedience. "The pulpits," says Lecky, "rang with declamations about the danger of the Church, with invectives against Nonconformists, with covert attacks upon the (Whig) Ministers."

A rector of St. Saviour's, Southwark, became the "hero of a drama," the famous Dr. Sacheverell, whose sermon, "On the Perils from False Brethren," was preached on November 5th, 1709, in St. Paul's Cathedral, before the Lord Mayor and the aldermen of London.

Sacheverell was impeached at the Bar of the House of Lords, and in consequence his name became a party cry throughout the length and breadth of England.

Prayers were offered up in the Royal Chapel itself for "Dr. Sacheverell under persecution." Multitudes thronged to kiss the doctor's hand as he passed through the streets. "Matters of Government and affairs of State," writes Defoe, "now became the province of the ladies" . . . . "they have hardly leisure to live, little time to eat and sleep, and none at all to say their prayers. . . . Little Miss has Dr. Sacheverell's picture put into her prayer book, that God and the doctor may take her up in the morning before breakfast."

We may rest assured that the ladies of Southwark Borough were not less excited than the rest of the world concerning the fate of their pastor. When women are roused to interest in any question they are always in earnest about it, and a nurse of Guy's Hospital in 1894 will be no exception to the rule. For the sake of her chosen profession she must dream of the Augustan age of Anne, with her face turned towards the hospital gates, and, if she be wise, she will not only refrain her tongue from talking "shop" during her two hours off duty, but will also carefully restrain her imagination from dwelling upon romantic Southwark when cutting dressings for the use of patients in the wards of Guy's Hospital.

### Notes from Holland.

The society which aims at giving instruction in sick nursing at the Hague is under the presidency of Her Majesty the Queen Regent, who takes much interest in its development. The Deaconesses' Institute at Arnheim, which was opened in May in the presence of a large assemblage of guests, has been greatly enlarged and rebuilt. Great interest has been taken in the improved structural arrangements, the wards being now particularly bright and well furnished. Those on the first and second storeys have verandahs, giving the patients pleasant resting places, into which they are moved in their beds when unable to leave them.

The deaconesses have large dining and reading rooms, and the patients have an excellent parlour. There is a good chapel, a steam laundry, and a kitchen with every modern convenience.

One hundred and forty-eight children were received into the Children's Hospital at Utrecht during last year, but unfortunately the financial condition of this institution is not so favourable as it might be.

The duties of the deaconesses and their hours of work are arranged by the Mother, or "head" of the institute. Each is entitled to an annual holiday, and has every Sunday free except when engaged with a case of very serious illness.

The municipal body, society, or persons who require the services of a deaconess, pay to the institute the sum of 250 francs a year, undertaking to provide the deaconess with good food and all other necessaries whilst the engagement lasts.

☛ For Everybody's Opinion, Reading, to the Sick, Book World for Women and Nurses, &c., see page cxxxix. et seq.

### Everybody's Opinion.

[Correspondence on all subjects is invited, but we cannot in any way be responsible for the opinions expressed by our correspondents. No communications can be entertained if the name and address of the correspondent is not given, or unless one side of the paper only be written on.]

### THE "NURSING RECORD" ON "POETRY."

"F.R.C.S." writes: Your contemporary, the *Nursing Record*, has made an unwonted excursion into the realm of literature, and has brought from thence a "beautiful poem," which "should prove interesting to nurses." With this prefatory statement, and with some account of the history of the "poem" in question, or rather of so much of that history as has "transpired"—("transpired" is good!)—the *Record* presents its readers with a grotesque paraphrase of the last four stanzas of some lines "On a Skeleton," which have done duty for more than fifty years in collections of minor sacred verse, and which, being by no means devoid of merit of a certain order, are deservedly held in high esteem by large numbers of Sunday-school teachers and children. As published in the *Record*, the lines are as follows; and without counting the mere misuse of capital letters, the four stanzas contain twenty-four errors which affect either the sense or the grammar, and which are indicated by printing them in italics:—

THE "NURSING RECORD" VERSION.

Beneath this mouldering canopy
Once shone *a* bright and busy eye:
But start not at the dismal void,
If *social* love that eye employed,
If with no lawless fire*s* it gleamed,
But through the dew*s* of kindness beamed,
That eye shall be for ever bright
When sun and stars *are sunk in night.*

*Within* this *hollow* cavern hung
The ready, swift, and tuneful tongue:
If falsehood's honey it disdained,
And where it could not praise, was chained;
If bold in virtue's cause it spoke,
Yet gentle concord never broke;
*This silent* tongue shall plead for thee,
When *time* unveils eternity.

Say, did these fingers *delve* the mine,
Or with its envied rubies shine?
To hew the rock *and* wear the gem
Can *little* now avail to them.
But if the *path* of *t*ruth they sought,
Or comfort to the mourner brought,
These hands a richer mead shall claim
Than all that *wait* on *W*ealth or *F*ame.

Avails it whether bare or shod
These feet the *path* of duty trod:
If from the bowers of *ease* they fled
To *seek* affliction's humble bed;
If *G*randeur's guilty bribe they spurned,
And home to *V*irtue's *cot* returned,
These feet with angel wings shall *rise*
And tread the palace of the *skies*.

I do not think that any commentary upon this rigmarole can be required, but the occasion is tempting. The substitution of "social" for "Christian" is perhaps not unworthy of notice as the most flagrant possible departure from the thought and intention of the original. "*Stars* sunk in *night*," the time at which they are most brilliant, as a metaphor for their extinction, is distinctly funny; and it is at least superfluous to describe a "cavern" as "hollow." The idea of the "silent" tongue "pleading" must have been stolen from Tennyson by a plagiarist too dull to perceive that his lines,

"Sorra the dhry eye thin but was wet for the frinds that was gone!
Sorra *the silent throat but we hard it cryin'* '*Ochone!*'"

although they possess a perfect fitness where he has placed them, in the mouth of an Irish peasant, would constitute a ludicrous blunder in any other connection. The idea of "time" *unveiling* "eternity," the use of the verb "to delve" without the preposition "into," and the suggestion that the "fingers" may have "sought" the "path" of "truth," while the feet "trod" the "path of duty," are perhaps the only other errors to which I need call attention. The numerous remaining ones will be rendered sufficiently conspicuous by comparing the *Record* version of the "poem" with the true one, which is here subjoined:—

THE ACTUAL POEM.

Behold this ruin! 'twas a scull,
Once of ethereal spirit full;
This narrow cell was life's retreat,
This space was thought's mysterious seat.
What beauteous pictures filled this spot,
What dreams of pleasure, long forgot!
Nor love, nor joy, nor hope, nor fear,
Has left one trace or record here.

Beneath this mouldering canopy
Once shone the bright and busy eye;
But start not at the dismal void—
If Christian love that eye employ'd,
If with no lawless fire it gleam'd,
But thro' the dew of kindness beam'd,
That eye shall be for ever bright
When stars and suns have lost their light.

Here in this silent cavern hung
The ready, swift, and tuneful tongue.
If falsehoods honey it disdain'd,
And where it could not praise, was chain'd,
If bold in virtue's cause it spoke,
Yet gentle concord never broke,—
That tuneful tongue shall plead for thee
When death unveils Eternity.

Say, did these fingers dig the mine?
Or with its envied rubies shine?
To hew the rock, or wear the gem,
Can nothing now avail to them:
But if the page of Truth they sought,
Or comfort to the mourner brought,—
These hands a richer meed shall claim
Than all that waits on wealth or fame.

Avails it whether bare or shod
These feet the paths of duty trod?
If from the bowers of joy they fled,
To soothe affliction's humble bed;
If grandeurs guilty bribe they spurn'd,
And home to virtue's lap return'd,
These feet with angel's wings shall vie
And tread the palace of the sky.

### A CORRECTION.

MR. C. BULLOCK, L.R.C.P., writes: Perhaps you will excuse my pointing out to you several inaccuracies in what would otherwise be useful and interesting articles on "The Mechanism of Movement" in the "Mirror" of June 23rd and 30th. For instance: "Movements of lower arm due to shoulder-joint;" "Sliding of bones of vertebræ upon one another," &c., in the issue for June 23rd. "Spinal column in twenty-four distinct pieces," &c., in issue for June 30th. The foregoing statements are certainly incorrect, and therefore I hope you will excuse the liberty I have taken of pointing them out to you, as such an article should be very useful to persons who are engaged in nursing.

*** As regards the points in the articles on "Mechanism of Movement," to which attention is called, the first, attributing the movements of the forearm to the shoulder-joint, was clearly an error, and should have been "the elbow-joint." As regards the second point, it seemed to me that the movement between the vertebræ themselves could best be described as "sliding," by which I refer to the movement allowed by the Multifidus Spinæ muscle, the action of which is described by Gray as "preserving the erect position and serving to rotate it." As regards the twenty-four "distinct vertebræ," these were mentioned as being those which contribute to the movements of the spinal column, and which are described as remaining "separate in the adult, and retaining their mobility." I did not intend to describe the spinal column as a whole, and hence omitted to enumerate all the thirty-three vertebræ which form it. Throughout these articles it has not been my aim to write a detailed treatise on anatomy, but merely to draw attention to practical points that might interest and help nurses, and I am glad to have had my attention called to the points in question.—The Writer of the Articles.

### EDUCATION OF A CRIPPLE.

A Matron writes: Will one of the numerous readers of The Hospital help me with advice regarding a little cripple? He is quite intelligent, and is now eleven years of age, and requires to be educated to get his own living. He might become a clerk or accountant if well taught, but of course the requisite instruction cannot be obtained for him in an ordinary home or hospital. I shall therefore feel greatly indebted to anyone who will tell me of a boarding school or institution in London where for quite moderate terms the boy could be educated.

### DISTRICT NURSING.

Dr. Christian Simpson writes from Tunbridge Wells: Perhaps I may be allowed to make a few remarks on the subject of district nurses and nursing. I am glad of this opportunity to testify my complete satisfaction and gratitude to the staff of district nurses working in this town. It is perfect nonsense to think that a three months' probationer is fit to do this class of work. It is likewise nonsense to think that a poor woman prefers a woman of her own class and station to nurse her in illness. The amount of tact and good nature required is far above that which is necessary in hospital, as a word loosely spoken, fussy interference with purely domestic arrangements, or a dozen other deeds of omission and commission, all done in perfect innocence, perhaps, makes all the difference between success and retaliation and repugnance to "having a nurse in the house." During the past twelve months I have had many serious medical and surgical cases among the poor, and all have required the utmost care and attention, which are derived from experience alone, and which it would be out of the question to expect from a probationer of the upper servant class. It is the greatest help to the doctor to have a reliable note of what has happened since his last visit, and likewise a great satisfaction to know that his orders will be carried out by a competent nurse in his absence. I think Miss Mary Mason cannot congratulate herself on the expression of her crude opinions on a matter of the utmost importance to the poor, and to the doctors who have to attend them. There is a daily increasing number of patients who prefer to have operations at home rather than go to an hospital, and this system of district nursing is the only adequate one to meet the legitimate desire of poor people. Only those who know can appreciate the difference in the state of a patient and the home during an illness when a competent district nurse is, or is not, in attendance.

### "The Hospital" Convalescent Fund.

We have received a donation of £1 for this Fund from Sister Edith, which we acknowledge with many thanks. Nurses will be glad to hear that through the funds they have subscribed, several nurses have been enabled to spend a delightful time at the sea, regaining health and strength for their work. At the close of the summer we propose to give our subscribers a short narrative of the help they have rendered.

### For Reading to the Sick.

**Motto.**

Christ's whole life was a Cross and a Martyrdom:
And dost thou seek rest and joy for thyself?
—*Thomas à Kempis.*

**Verses.**

Faith needs no staff of flesh, but stoutly can
To heaven alone both go and lead.
—*George Herbert*

God loves to work in wax—not marble.
Let Him find
When He would mould thine heart,
Material to his mind.—*Trench.*

Peace! Peace!
Wrought by the spirit of might,
In thy deepest sorrow and sorest strife,
In the changes and chances of mortal life—
It is thine, beloved! Christ's own bequest,
Which vainly the tempter shall strive to wrest,
It is now thy right! —*F. R. Havergal.*

Yield to the Lord, with simple heart,
All that thou hast, and all thou art!
Renounce all strength but strength Divine,
And peace shall be for ever thine.
—*Mdme. Guion.*

**Reading.**

What was St. Paul's "thorn in the flesh?" It is easy at once to guess that it was not an actual real thorn that is meant. No, it is but a figure or image to express a sharp painful thing. Some ailment not of soul, but of body.

St. Paul might have told us if he would what the thorn really was. But it was most likely on purpose that he calls his ailment by this figurative, and not by its real name. For now all who suffer (and there are many different ways of suffering) may learn the lesson that he learnt. Each one may, if he will, call his particular trial "a thorn in the flesh" just as St. Paul did.

There is probably one special reason why he calls it a thorn, because, when a thorn is drawn out there is relief at once. The flesh directly begins to heal, because the cause of the pain and irritation is gone. In a moment it is done and all is well.

St. Paul's "thorn" was something like this. It was capable of being cured, he thought. Probably it was some kind of chronic malady, which was a constant burden and drawback to enjoyment, and a continual drag on the powers of usefulness. Such trials do not always excite much pity, for it is supposed the sufferer "gets used" to them. So he does, perhaps, but time does not make the burden materially lighter. It *is* a trial, and will be one to the end. St. Paul did the best thing to do in any trouble, he prayed to God about it. He prayed earnestly that it might be taken away. Although he prayed his prayer three times that the "thorn" might depart there was no sign of cure. Was there then no answer? Yes, in the quiet hours of the night, perhaps, some words were borne in upon his mind, "My grace is sufficient for thee, My strength is made perfect in weakness," and they are a full and complete answer, if only we think about them a little.

We are sure God heard the prayer. And He who heard it could have granted it. But He withholds His hand from doing so, and hence we gather that there must have been a reason and purpose in the affliction. It was no blind chance that sent it. And the purpose was that God's strength might be made perfect in the weakness of His servant . . . That so by degrees the world might approach the truth, that it was the power of Christ that filled the feeble body. It was that which wrought in him, and caused him to lose himself in Christ. "Not I, but Christ liveth in me." Men would see at last that he was but an instrument in the hands of the Mighty Worker, the weak man made perfect by Divine strength.

—*C. M. Hallett.*

### The Book World for Women and Nurses.

[We invite Correspondence, Criticism, Enquiries, and Notes on Books likely to interest Women and Nurses. Address, Editor, The Hospital (Nurses' Book World), 428, Strand, W.C.]

I Forbid the Banns. By Frank Frankfort Moore. (London: Hutchinson and Co. 1893.)

This is a decidedly clever and interesting story. The dialogue is witty enough in itself to be readable, quite apart from the plot, which is most daring in conception, if not entirely original. Bertha Lancaster makes a charming heroine; a beautiful Australian, possessing some thousands per annum, cannot fail to be so, especially when imbued as she is here shown with advanced and emancipated ideas. Julian Carlton, a more ordinary type, a well-to-do country gentleman of moral worth and fair intellect falls madly in love with her on board ship, homeward bound. His love is returned, but at this point the heroine's notions come in rather awkwardly, and Bertha absolutely refuses to go through the marriage ceremony, which she denounces as "horrible and slavish," "that legalized compulsory union," as she styles it. Carlton is too much of a man of the world not to realize that such principles—based even on a pure foundation—won't work, and he bravely holds out for some time, but finding that he cannot obtain possession of his love by legal means, submits, at the risk of scandal that must unavoidably follow. The description of their reception in an old-fashioned country society is humorous, when not pathetic. However, it is in London the real fiasco takes place, pangs of jealousy seize the husband—in name only; and after bitter words a separation ensues. This, of course, ends in a re-union, a collapse of the theories of modern sociology and woman's emancipation, which is duly followed by a prosaic marriage. Yet our sympathies are with them to the end, and we are thankful to leave such a couple in peace, and respectably re-established in the eyes of the world. The subsidiary characters are also well drawn by the author, Marian Travers, a revengeful, jealous woman, and Cyril Southcote, a specimen of real educated priggishness, being very true to nature. Perhaps the characters of Sir Ecroyd Fairleigh, and Eric Vicars, the would-be-lovers of Bertha Lancaster, are somewhat exaggerated, but help to make complete an extremely good tale.

Handbook of Obstetric Nursing. By Haultain and Ferguson. Second edition. (Young J. Pentland. Edinburgh and London.)

This clearly-written and practical book contains useful information and guidance for those engaged in nursing puerperal cases. The remarks on the necessity of absolute cleanliness in every detail of dress and person of the nurse are excellent, also the suggestions for the antiseptic treatment of patients and appliances. Nevertheless, the recommendation of turpentine for cleansing the hands is rather a severe measure, and might lead to roughness and abrasions of the skin, which would prove undesirable. The value of carbolised oil also, even when diluted to a strength of 1 in 20, is now questioned, and strictly antiseptic carbolised or mercurialised vaseline is usually recommended instead. The chapters dealing with the special anatomy, physiology, and phenomena of gestation are simply and clearly written, so that anyone can form a good general idea of the subject; the diagrams are a great help in this. Chapter IX. is specially to be recommended to monthly nurses. The management of normal labour is clearly laid down, with many valuable practical details. Exception might be taken to the administration of ergot at the end of the second instead of the third stage of labour. Also it is not clearly stated if the monthly nurse is expected to make internal examinations, or if these directions only refer to cases undertaken by a midwife. The phenomena of abnormal and complicated labour are concisely stated, and due emphasis is laid on the absolute necessity of sending at once for skilled medical aid if anything unusual occurs. Far better for the nurse to incur the wrath of "the busy doctor, roused in the early hours of the morning," than any complication should be allowed to assume a serious aspect before help is summoned. The question of hæmorrhage is not very fully treated, a most important one for both midwife and nurse; nor are mechanisms fully described, and the complications connected with them, which latter need equally prompt recognition on the part of a midwife to avoid loss of time in procuring medical assistance. In Chapter XI. an impression is given, unintentionally of course, that the management of such cases might be undertaken by the nurse without a doctor being called in at the time. Nothing in the least abnormal should ever be thought within the range of anyone except a qualified practitioner. The aftercare of mother and infant, and the "description of certain methods and appliances," are instructive, and eminently practical and helpful. The book is well worth studying, and will be found very useful to obstetric nurses.

---

### MAGAZINES OF THE MONTH.

"The Decline and Fall of Napoleon" is continued in this month's Pall Mall Magazine, for which alone, were it for nothing else, the present issue is well worth reading. Lord Wolseley deals here with the period of the "Hundred Days" and the consequent battle of Ligny. "Napoleon's return to France was not influenced," his biographer remarks with an unflinching regard to truth, "by any patriotic motive, but it was the outcome of a fiendish and inordinate ambition of the most selfish kind. It meant a new outburst of war, more bloodshed, and a fresh crop of misery to Europe. France required peace above all things after her many years of revolutionary horrors and devastating strife; but Napoleon brought her war with England and every Continental State. His return begat new trials and new sufferings for humanity." Perhaps of all the aspects of the great Napoleon's life this epoch is the most interesting, and Lord Wolseley deals with it in a masterly manner.

The English Illustrated Magazine for July contains much excellent matter. "The Return of the Probationer," for instance, a short story by Mr. E. F. Benson, is both interesting and well written. Mr. Benson is well known to the literary public, but he shows himself in somewhat a new light in the present instance. The tale is unlike the author's longer works, and what recommends it especially is the absence of a melancholy and morbid spirit which characterise much of the literature of the latter half of the 19th century. There are some charming illustrations in this month's magazine, and a well-written article on tapestry, dealing with the part history of the industry.

The July number of the Cornhill Magazine gives us the opening chapters of a new serial story which promises to sustain our interest. It is written by the author of "The Poison of Asps," Mr. R. O. Prowse; the present serial is called "A Fatal Preservation." There is always some serious as well as good fictional matter in the Cornhill, and the present issue has proved no exception. "With R. L. Stevenson in Samoa," for instance, is delightful reading, as indeed is also James Payn's "Gleams of Memory."

Cassells Family Magazine adapts itself with a wide catholicity to all kinds and conditions of men. It has its educational and its romance side, and the present issue sustains its character in an admirable manner. The July number, besides having some good fiction, contains a short but nteresting article by Sir Robert Ball on "The Pleiades."

